# Supplementary material for: Regulation of therapeutic protein release in response to circadian biomarkers
Source: Nat Commun. 2025 Nov 6;16:9812. doi: 10.1038/s41467-025-64761-9 (PMC12592386; doi:10.1038/s41467-025-64761-9)
Supplement: Supplementary file 1 — Supplementary information [file 41467_2025_64761_MOESM1_ESM.pdf]

Supplementary Information

Regulation of therapeutic protein release in response to circadian biomarkers

Nik Franko[1], Shichao Li[2], Silvia Galvan[1], Zsoka Csorba[1], Ana Palma Teixeira[1], Mingqi Xie[2], Martin Fussenegger[1,3,*]

[1]Department of Biosystems Science and Engineering, ETH Zurich, Klingelbergstrasse 48, CH-4056 Basel, Switzerland.

[2]Westlake Laboratory of Life Sciences and Biomedicine, Hangzhou, Zhejiang, China.

[3]Faculty of Science, University of Basel, Klingelbergstrasse 48, CH-4056 Basel, Switzerland.

[*]Correspondence: martin.fussenegger@bsse.ethz.ch

Contents

1. **Supplementary Figures 1-5**
2. **Supplementary Table 1**

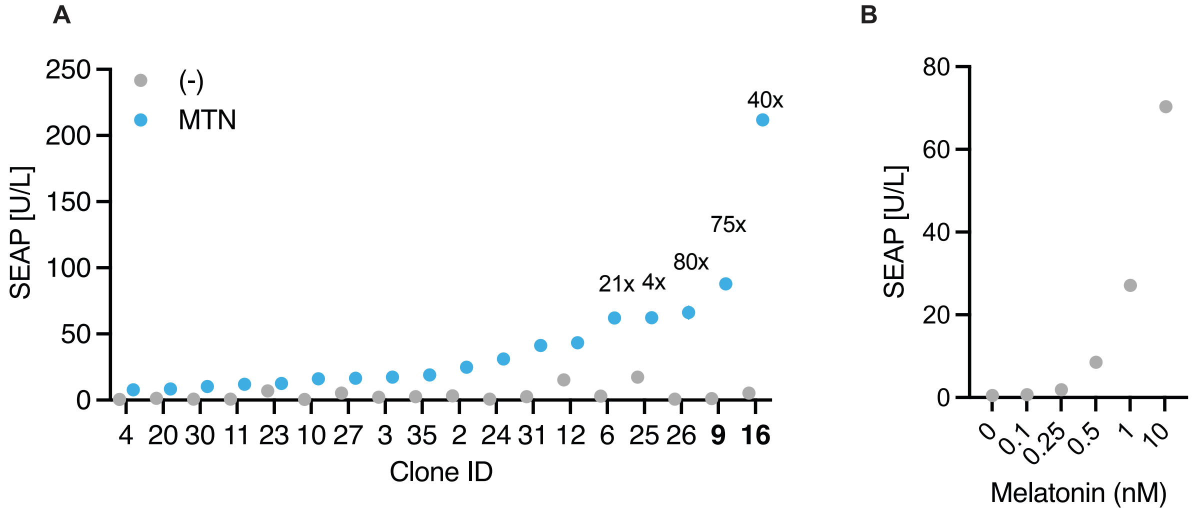

Supplementary Fig. 1. a Primary and **b** secondary screening (Clone 9) of melatonin-responsive cell clones. Monoclonal cell lines derived from a population of cells with stably integrated pNF394 and pNF396 were cultivated in the absence or presence of melatonin (10 nM) for 24 h before profiling SEAP expression. Clone with the highest SEAP expression capacity was cultured in the presence of different melatonin concentrations for 24 h before determining SEAP secretion levels. Data are shown as mean of *n* = 2 biological replicates. Numbers above the dots indicate fold induction between MTN-treated and un-treated cells. Source data are provided as a Source Data file.

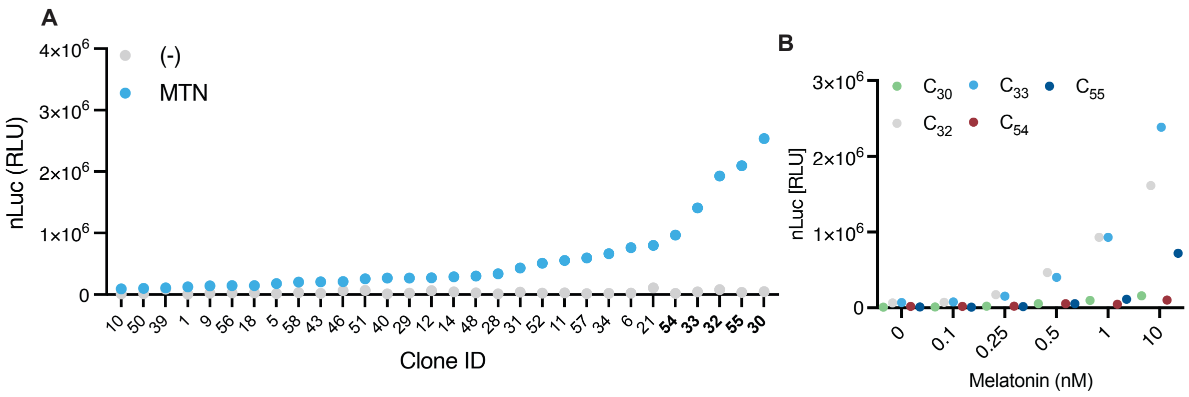

Supplementary Fig. 2. a Primary screening and **b** secondary screening of melatonin-responsive cell clones. Monoclonal cell lines derived from a population of cells with stably integrated pNF395 and pNF396 were cultivated **a** in the absence or presence of melatonin

(10 nM) or **b** with indicated MTN levels for 24 h before profiling nLuc expression. Data are shown as mean of n = 2 biological replicates. Source data are provided as a Source Data file.

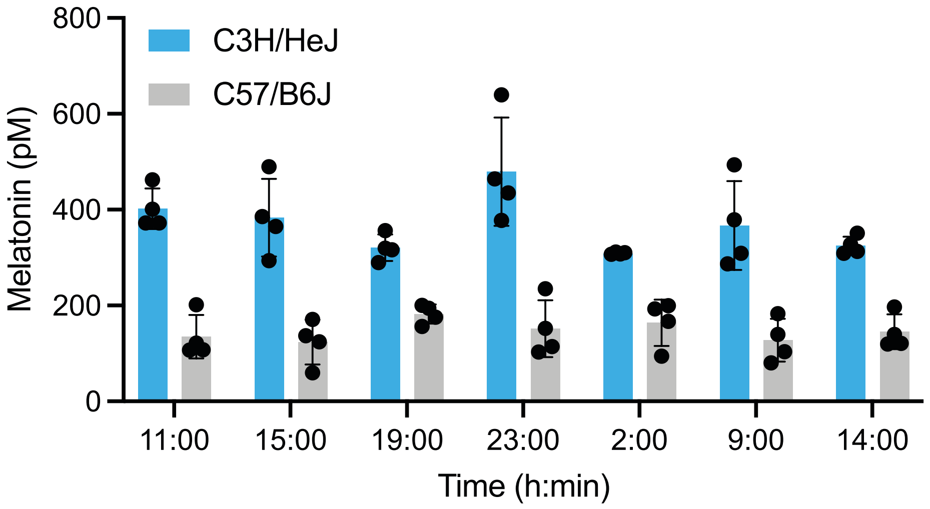

Supplementary Fig. 3. Time-course of blood melatonin levels in mice. Serum of male C3H/HeJ or C57BL/6J mice were collected at different timepoints of an experimental day for quantification of blood melatonin levels using a cell-based assay described in Supplementary Fig. 4. Data are shown as mean ± SD of n = 4 biological replicates. Source data are provided as a Source Data file.

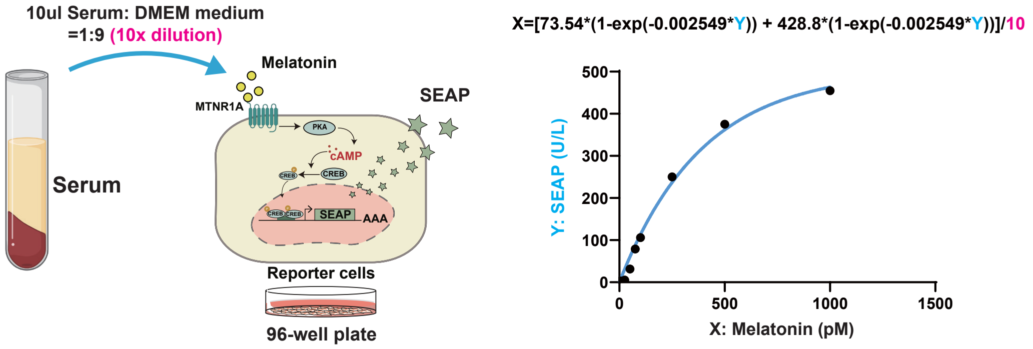

Supplementary Fig. 4. Quantification of melatonin levels using a custom-designed reporter assay. To detect melatonin levels in culture supernatants or animal serum, reporter cells were engineered by co-transfection of a constitutive MTNR1A expression vector (pNF396, 90ng) and a P_{CRE}-driven SEAP expression vector (pDJ667, 30ng), followed by stimulation with 10%

(v/v) of heterogenous melatonin-containing samples. At 48h after stimulation, semi-quantitative melatonin levels in assay samples were estimated by comparing SEAP levels in culture supernatants with SEAP levels produced by the same reporter cells cultivated in medium supplemented with known melatonin concentrations. Data are shown as mean of n=3 independent experiments. Source data are provided as a Source Data file.

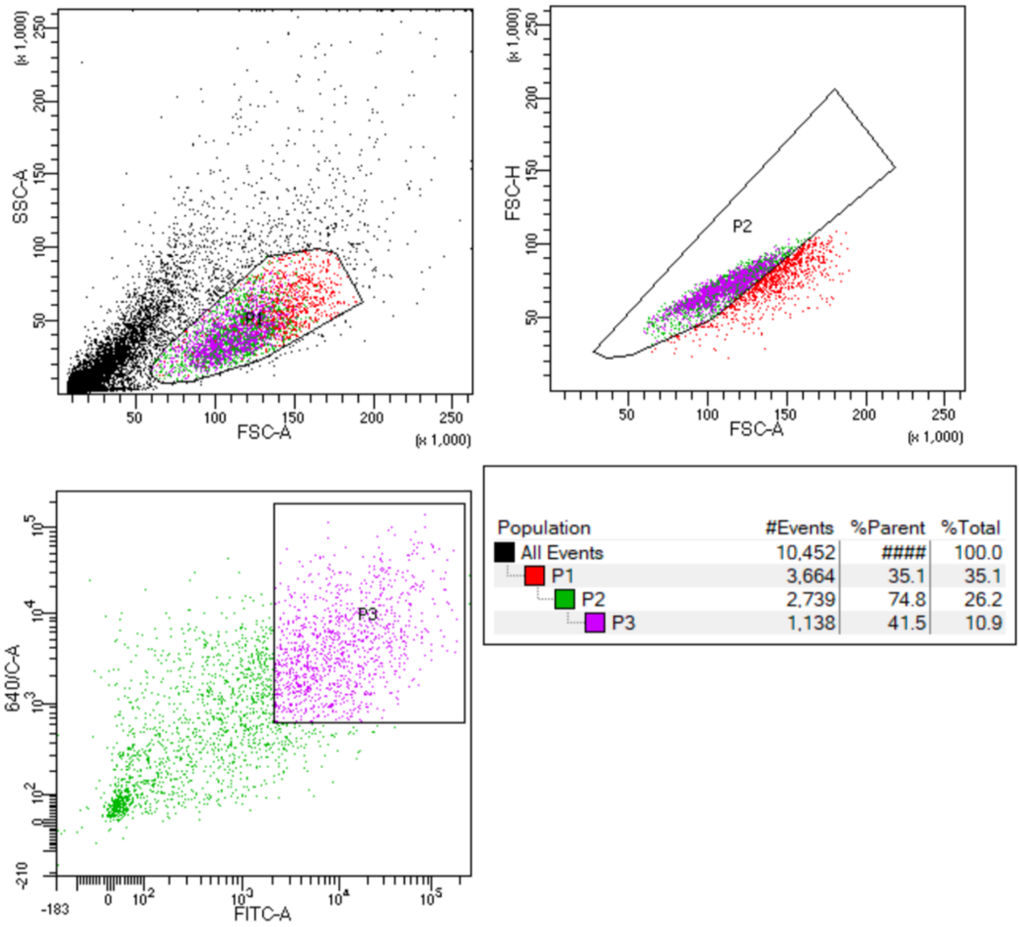

Supplementary Fig. 5. Gating strategy for sorting engineered HEK293T cells double positive for YPet and iRFP. Antibiotic-selected live (P1) cells (FCS-A vs. SSC-A) were gated (P2) for singlets (FSC-A vs. FSC-H), followed by selection of YPet/iRFP double positive (P3) cells (FITC-A vs. 640/C-A).

Supplementary Table S1. Sequences of CRE response regions (CRE response elements + minimal promoter) upstream of a reporter gene.

Reporter	Sequence
pCK53	GCACCAGACAGTGACGTCAGCTGCCAGATCCCATGGCCGTCATACTGTGACGTCTTTCAGACACCCCATTGACGTCAATGGGAGAACAGATCTGCCGCCCCGACTGCATCTGCGTGTTCGAATTCGCCAATGACAAGACGCTGGGCGGGGTTTGTGTCATCATAGAACTAAAGACATGCAAATATATTTCTTCCGGGGACACCGCCAGCAAACGCGAGCAACGGGCCACGGGGATGAAGC
pSP16	GCTAGCAGCCTGACGTCCGAGAGCCTGACGTCCGAGAGCCTGACGTCCGAGAGCCTGACGTCCGAGATCTCTCGAGGTCGACAGCGGAGACTCTAGAGGGTATATAATGGAAGCTCGACTTCCAGCTTGGCAATCCGGTACTGTTGGTAAAGAATTCACC
pVH421	AGCCTGACGTCCGAGAGCCGTAGCCTGACGTCCGAGGGTACCAGCCTGACGTCCGAGAGCCGTAGCCTGACGTCCGAGTACTCCGTAGAGGGTATATAATGGAAGCTCGACTTCCAGCTTGGCGAGCTCTTCGAAGCGGAATTCACC

Color legend:

consensus CRE-operator site

TATA-like minimal promoter

non-consensus CRE-operator site

minP promoter
