# Supplementary material for: Regulation of therapeutic protein release in response to circadian biomarkers
Source: Nat Commun. 2025 Nov 6;16:9812. doi: 10.1038/s41467-025-64761-9 (PMC12592386; doi:10.1038/s41467-025-64761-9)
Supplement: Supplementary file 3 — Description of Additional Supplementary Files [file 41467_2025_64761_MOESM3_ESM.pdf]

Description of Additional Supplementary Files

File Name: Supplementary Data 1
Description: Plasmids used and designed in this study. Plasmids that have been constructed for this study contain a detailed description of a cloning procedure.
