# Supplementary material for: Regulation of therapeutic protein release in response to circadian biomarkers
Source: Nat Commun. 2025 Nov 6;16:9812. doi: 10.1038/s41467-025-64761-9 (PMC12592386; doi:10.1038/s41467-025-64761-9)
Supplement: Supplementary file 5 — Reporting Summary [file 41467_2025_64761_MOESM5_ESM.pdf]

nature portfolio

Corresponding author(s): Prof. Dr. Martin Fussenegger

Last updated by author(s): Jul 12, 2025

Reporting Summary

Nature Portfolio wishes to improve the reproducibility of the work that we publish. This form provides structure for consistency and transparency in reporting. For further information on Nature Portfolio policies, see our Editorial Policies and the Editorial Policy Checklist.

Statistics

For all statistical analyses, confirm that the following items are present in the figure legend, table legend, main text, or Methods section.

n/a	Confirmed	
☐	☒	The exact sample size (*n*) for each experimental group/condition, given as a discrete number and unit of measurement
☐	☒	A statement on whether measurements were taken from distinct samples or whether the same sample was measured repeatedly
☐	☒	The statistical test(s) used AND whether they are one- or two-sided<br>*Only common tests should be described solely by name; describe more complex techniques in the Methods section.*
☒	☐	A description of all covariates tested
☐	☒	A description of any assumptions or corrections, such as tests of normality and adjustment for multiple comparisons
☐	☒	A full description of the statistical parameters including central tendency (e.g. means) or other basic estimates (e.g. regression coefficient) AND variation (e.g. standard deviation) or associated estimates of uncertainty (e.g. confidence intervals)
☐	☒	For null hypothesis testing, the test statistic (e.g. *F*, *t*, *r*) with confidence intervals, effect sizes, degrees of freedom and *P* value noted<br>*Give P values as exact values whenever suitable.*
☒	☐	For Bayesian analysis, information on the choice of priors and Markov chain Monte Carlo settings
☒	☐	For hierarchical and complex designs, identification of the appropriate level for tests and full reporting of outcomes
☒	☐	Estimates of effect sizes (e.g. Cohen's *d*, Pearson's *r*), indicating how they were calculated

Our web collection on statistics for biologists contains articles on many of the points above.

Software and code

Policy information about availability of computer code

Data collection	Luminescence and absorbance data were collected with Tecan Spark plate reader (TECAN AG).
Data analysis	The data were analyzed using Prism GraphPad 9 (GraphPad Prism software Inc.) and Microsoft Excel for iOS (version 16.92).

For manuscripts utilizing custom algorithms or software that are central to the research but not yet described in published literature, software must be made available to editors and reviewers. We strongly encourage code deposition in a community repository (e.g. GitHub). See the Nature Portfolio guidelines for submitting code & software for further information.

Data

Policy information about availability of data

All manuscripts must include a data availability statement. This statement should provide the following information, where applicable:

- Accession codes, unique identifiers, or web links for publicly available datasets
- A description of any restrictions on data availability
- For clinical datasets or third party data, please ensure that the statement adheres to our policy

The authors declare that all data supporting the findings of this study are available within the paper, Supplementary Information and the Source Data file. All plasmids used in this study are listed in Supplementary Data 1 and are available upon request from the corresponding author. Source data are provided with this paper.

Research involving human participants, their data, or biological material

Policy information about studies with human participants or human data. See also policy information about sex, gender (identity/presentation), and sexual orientation and race, ethnicity and racism.

Reporting on sex and gender	N/A
Reporting on race, ethnicity, or other socially relevant groupings	N/A
Population characteristics	N/A
Recruitment	N/A
Ethics oversight	N/A

Note that full information on the approval of the study protocol must also be provided in the manuscript.

Field-specific reporting

Please select the one below that is the best fit for your research. If you are not sure, read the appropriate sections before making your selection.

[x] Life sciences [ ] Behavioural & social sciences [ ] Ecological, evolutionary & environmental sciences

For a reference copy of the document with all sections, see nature.com/documents/nr-reporting-summary-flat.pdf

Life sciences study design

All studies must disclose on these points even when the disclosure is negative.

Sample size	No statistical methods were used to predetermine sample size. Sample size was determined based on similar studies in our lab and other published studies in our field (Scheller L, Nat. Commun. 2020; Krawczyk K, Nat. Commun. 2020). n=3 biologically independent samples were predicted to be sufficient for detecting statistically relevant differences between compared groups in cell culture experiments.
Data exclusions	All data was included.
Replication	Attempts at replication were successful, with each experiment replicated at least 3 times. Detailed replication times are listed in each figure legend.
Randomization	For the mouse studies, animals of the same genetic background were randomly allocated into different experimental groups. For cell culture experiments, no covariates based on sample allocations to experimental groups could be observed and no randomization was performed. All direct comparison of induced vs. non-induced were performed with cells transfected under the same conditions with the same transfection mixture.
Blinding	The investigators were not blinded to allocation during experiments and outcome assessment. Blinding was not possible as the same investigator processed the animal/cell culture experiments and analyzed the data.

Reporting for specific materials, systems and methods

We require information from authors about some types of materials, experimental systems and methods used in many studies. Here, indicate whether each material, system or method listed is relevant to your study. If you are not sure if a list item applies to your research, read the appropriate section before selecting a response.

Materials & experimental systems

n/a	Involved in the study	
[x]	[ ]	Antibodies
[ ]	[x]	Eukaryotic cell lines
[x]	[ ]	Palaeontology and archaeology
[ ]	[x]	Animals and other organisms
[x]	[ ]	Clinical data
[x]	[ ]	Dual use research of concern
[x]	[ ]	Plants

Methods

n/a	Involved in the study	
[x]	[ ]	ChIP-seq
[ ]	[x]	Flow cytometry
[x]	[ ]	MRI-based neuroimaging

Eukaryotic cell lines

Policy information about cell lines and Sex and Gender in Research

Cell line source(s)	Human embryonic kidney cells (HEK-293, ATCC: CRL-11268), adipose tissue-derived human telomerase reverse transcriptase-immortalized human mesenchymal stem cells (hMSC-hTERT, ATCC: SCRC4000), Golden hamster kidney fibroblasts (BHK-21, ATCC: CCL-10), Human adenocarcinoma cell line (HeLa, ATCC: CCL-2), Chinese hamster ovary cells (CHO-K1, ATCC: CCL61).
Authentication	Cells were authenticated by ATCC and no further authentication was performed. The quality and phenotype of the cells was routinely checked by microscopy.
Mycoplasma contamination	The authors confirm that the cell lines in this study were tested for mycoplasma negative.
Commonly misidentified lines (See ICLAC register)	Cell lines used in this study are not listed in the ICLAC register as commonly misidentified lines.

Animals and other research organisms

Policy information about studies involving animals; ARRIVE guidelines recommended for reporting animal research, and Sex and Gender in Research

Laboratory animals	8-week-old male C57BL/6J mice were obtained through the Laboratory Animal Resources Center (LARC) of Westlake University. 8-week-old male BKS. Lepr (db/db) mice were obtained from Shanghai Institutes for Biological Sciences Shanghai Laboratory Animal Center (SLACCAS) (Shanghai, China). 8 week-old male C3H/HeJ mice were obtained through the Laboratory Animal Resources Center (LARC) of Westlake University. Mice were housed under a 12-hour light–dark cycle, with five animals per cage. The ambient temperature was maintained at 21±1°C, with humidity at 50±10%. Euthanasia was performed by placing the mice in a carbon dioxide (CO_2) chamber without prior pre-charging, and 100% CO_2 was delivered by displacing 20% of the chamber volume per minute. After 10 minutes, euthanasia was confirmed by the absence of chest movement, palpable heartbeat, and responsiveness to toe pinch.
Wild animals	The study did not involve wild animals.
Reporting on sex	No sex-related analysis was performed.
Field-collected samples	The study did not involve field-collected samples.
Ethics oversight	Animal experiments were performed according to the protocol (Protocol ID: 20-009-XMQ and 25-013-XMQ) approved by the Institutional Animal Care and Use Committee (IACUC) of Westlake University and in accordance with the Animal Care Guidelines of the Ministry of Science and Technology of the People's Republic of China.

Note that full information on the approval of the study protocol must also be provided in the manuscript.

Plants

Seed stocks	The study did not involve the use of plants.
Novel plant genotypes	The study did not involve the use of plants.
Authentication	The study did not involve the use of plants.

Flow Cytometry

Plots

Confirm that:

- ☒ The axis labels state the marker and fluorochrome used (e.g. CD4-FITC).
- ☒ The axis scales are clearly visible. Include numbers along axes only for bottom left plot of group (a 'group' is an analysis of identical markers).
- ☒ All plots are contour plots with outliers or pseudocolor plots.
- ☒ A numerical value for number of cells or percentage (with statistics) is provided.

Methodology

Sample preparation	Briefly, 3.5x105 HEK-293T cells were cultured in 6-well plates for 24h. To establish the stable cell line, cells were co-transfected with 200 ng of pTS395, which encodes constitutively expressed Sleeping Beauty transposase SB100X, 1000 ng of pNF396 and either 1000 ng of pNF394 or 1000 ng pNF395. 24 hours post-transfection, cells were transferred to fresh medium containing puromycin (1 µg/ml) and blasticidin (10 µg/ml) for a three-day antibiotic selection process. Subsequently, the polyclonal population of genetically engineered cells was suspended in DMEM medium and sorted with a fluorescence activated cell sorting (FACS) system (BD Biosciences) based on the yPET (517/530) andi RFP (690/713) fluorescence signals. The double-positive cell population was isolated as single cells in 96-well plates and maintained in double antibiotic-containing selection medium. Following two weeks of clonal expansion, monoclonal cell lines were screened by the addition of MTN, and the most promising cell line was selected for subsequent experiments.
Instrument	Flow cytometry analysis was performed with a FACSAria Fusion Cell Sorter, Becton Dickinson, NewJersey, USA
Software	Flow cytometry data were analyzed using FACSAria Fusion Cell Sorter, Becton Dickinson, NewJersey, USA
Cell population abundance	Using fluorescence output, the double-positive (yPET (517/530) and iRFP (690/713) fluorescence signals) cell population was sorted as single cells in 96-well plates and maintained in double antibiotic-containing selection medium.
Gating strategy	Gating for positive cells was performed on HEK-293T cells expressing no fluorophore.

☒ Tick this box to confirm that a figure exemplifying the gating strategy is provided in the Supplementary Information.
